# The CpG island methylator phenotype is concordant between primary colorectal carcinoma and matched distant metastases

**DOI:** 10.1186/s13148-017-0347-1

**Published:** 2017-05-02

**Authors:** Stacey A. Cohen, Ming Yu, Kelsey Baker, Mary Redman, Chen Wu, Tai J. Heinzerling, Ralph M. Wirtz, Elpida Charalambous, George Pentheroudakis, Vassiliki Kotoula, Konstantine T. Kalogeras, George Fountzilas, William M. Grady

**Affiliations:** 1 Clinical Research Division, Fred Hutchinson Cancer Research Center, Seattle, WA 98109 USA; 2 Division of Oncology, University of Washington, Seattle, WA USA; 3 Clinical Statistics, Clinical Research Division, Fred Hutchinson Cancer Center, Seattle, WA USA; 4 College of Life Sciences, Hebei University, Baoding, Hebei People’s Republic of China; 5 STRATIFYER Molecular Pathology GmbH, Cologne, Germany; 6 Laboratory of Molecular Oncology, Hellenic Foundation for Cancer Research/Aristotle University of Thessaloniki, Thessaloniki, Greece; 7 Department of Medical Oncology, Ioannina University Hospital, Ioannina, Greece; 8 Department of Pathology, Faculty of Medicine, School of Health Sciences, Aristotle University of Thessaloniki, Thessaloniki, Greece; 9 Translational Research Section, Hellenic Cooperative Oncology Group, Data Office, Athens, Greece; 10 Aristotle University of Thessaloniki, Thessaloniki, Greece; 11 Division of Gastroenterology, University of Washington School of Medicine, Seattle, WA USA; 12 825 Eastlake Ave E, G4-830, Seattle, WA 98109 USA; 13 1100 Fairview Ave N, D4-100, Seattle, WA 98109 USA; 14 1100 Fairview Ave N, M2-B230, Seattle, WA 98109 USA; 15 Werthmann Str. Str. 1c, D-50935 Cologne, Germany; 16 University Campus, Building 17B, 540 06 Thessaloniki, Greece; 17 Niarchos Av, Ioannina, 455 00 Greece; 18 18 Hatzikonstanti Str, 115 24 Athens, Greece; 19 30 Kapetan Kotta Str, 552 36 Panorama, Thessaloniki Greece

**Keywords:** CIMP, DNA methylation, Colorectal cancer, Metastatic, Biomarker

## Abstract

**Background:**

The CpG island methylator phenotype (CIMP) in stage III colon cancer (CRC) has been associated with improved survival after treatment with adjuvant irinotecan-based chemotherapy. In this analysis, we determine whether CIMP status in the primary CRC is concordant with the CIMP status of matched metastases in order to determine if assessment of CIMP status in the primary tumor can be used to predict CIMP status of metastatic disease, which is relevant for patient management as well as for understanding the biology of CIMP CRCs.

**Methods:**

We assessed the CIMP status of 70 pairs of primary CRC and matched metastases using a CRC-specific panel of five markers (*CACNA1G*, *IGF2*, *NEUROG1*, *RUNX3*, and *SOCS1*) where CIMP positive was defined as 3/5 positive markers at a percent methylated reference threshold of ≥10%. Concordance was compared using the Fisher’s exact test and *P* < 0.05 was considered significant.

**Results:**

Sixty-nine of the pairs (98.6%) showed concordant CIMP status in the primary tumor and matched metastasis; five (7.0%) of the pairs were concordantly CIMP positive. Only one pair (1.4%) had divergent CIMP status, demonstrating CIMP positivity (4/5 markers positive) in the primary tumor, while the matched metastasis was CIMP negative (0 markers positive).

**Conclusions:**

CIMP status is generally concordant between primary CRCs and matched metastases. Thus, CIMP status in the primary tumor is maintained in matched metastases and can be used to inform CIMP-based therapy options for the metastases.

**Electronic supplementary material:**

The online version of this article (doi:10.1186/s13148-017-0347-1) contains supplementary material, which is available to authorized users.

## Background

An increased understanding of colorectal cancer (CRC) molecular alterations has resulted in the characterization of novel biomarkers that are prognostic and/or predictive for treatment response. However, with the recognition of the molecular heterogeneity of CRC, the characterization of potential biomarkers has also become more complex and demanding [1]. For instance, there is potential variability between the primary tumor and matched metastatic foci, between multiple metastatic sites in one patient (intertumoral heterogeneity), and within a tumor itself (intratumoral heterogeneity) [2–4]. Thus, for a biomarker to be widely clinically useful, the relevance of the status of a biomarker in both the primary tumor and metastatic disease sites needs to be understood. Gaining this understanding not only improves the accuracy of the biomarker for directing the management of metastatic disease but also advances our understanding of the molecular pathogenesis of CRC and its metastasis.

CRCs are thought to arise through distinct molecular pathways. One commonly used molecular classification scheme for characterizing these distinct subgroups of CRC involves determining whether the tumors are chromosomally unstable (chromosomal instability, CIN; also referred to as microsatellite stable (MSS)), microsatellite unstable (MSI) or have the CpG island methylator phenotype (CIMP). While likely an oversimplification of the underlying tumor biology, the CIN, MSI, and CIMP subgroup classifications do demonstrate differences in prognosis and treatment responses and, thus, there is clinical utility in using them for distinguishing an individual’s CRC features [5]. CIMP is characterized by an exceptionally high level of genome-wide aberrant DNA methylation in CpG island regions and is often identified using the methylation status of discrete sets of specific CpG loci [6–9]. A CIMP assay panel commonly used to identify a CRC as demonstrating CIMP is a validated five-gene panel developed by Weisenberger et al. [9]. Found in 10–20% of CRCs, CIMP-positive CRCs are often associated with *BRAF* mutations, MSI (or deficiency in expression of the mismatch repair (MMR) proteins), proximal colon location, and female gender [9]. Although earlier studies have not reached consensus on the clinical value of CIMP status to predict response to 5-fluorouracil (5-FU) chemotherapy [10–12], more recent exploratory analysis indicates that CIMP is associated with an enhanced response to adjuvant irinotecan-based therapy for stage III CRC [13]. As the goal of adjuvant therapy is to limit disease recurrence, it is important to understand whether determination of CIMP status in the primary tumor is relevant to the molecular profile of metastatic disease. Furthermore, the CIMP status of the primary tumor has the potential to be used to select therapy for patients with metachronous metastatic disease, which is a second reason to determine if the CIMP status in the primary tumor is also present in matched metastatic lesions.

As primary and metastatic tumor tissues may not both be clinically available, it is necessary to evaluate the concordance of CIMP across tissue sites. As has been previously demonstrated, key biomarkers (such as mutant *KRAS*) may be present in the primary tumor, but not the metastatic clones, or vice versa, and prior treatment can impact their presence in the recurrent tumor [14–16]. In this study, we examined the CIMP status of primary CRC and matched metastases to determine the concordance rate of CIMP status within primary tumor-metastasis pairs, accounting for key clinical factors.

## Methods

### Patient selection

Clinical data and formalin-fixed paraffin-embedded (FFPE) tumor tissue samples from 80 CRC patients with both primary and matched metastatic samples available were retrospectively retrieved from the Clinical Data Bank and the Tumor Repository of the Hellenic Cooperative Oncology Group (HeCOG). Patients had been treated in different Oncology departments in HeCOG-affiliated hospitals from 2006 to 2015. The study protocol and the informed consent form were approved by the Bioethics Committee of the Aristotle University of Thessaloniki School of Medicine (July 15, 2016) and were in agreement with the 1975 Helsinki statement (revised in 1983). Written informed consent was obtained from all patients for the use of their biological material for research purposes.

### DNA extraction

DNA and total RNA were extracted from 1.0 mm TMA cores (15–18 tumor tissue cores per sample), using a standardized fully automated isolation method based on germanium-coated magnetic beads (XTRAKT kit, STRATIFYER Molecular Pathology GmbH, Cologne, Germany) in combination with a liquid handling robot (XTRAKT XL, STRATIFYER Molecular Pathology GmbH), as previously described [17].

### Sodium bisulfite conversion and sample preparation

Genomic DNA from each sample was bisulfite converted using the EZ DNA Methylation Kit (ZymoResearch, Irvine, CA) according to the manufacturer’s instructions with an elution volume of 20 μL for MethyLight analysis.

### *KRAS*, *BRAF*, and mismatch repair analysis

*KRAS* and *BRAF* mutational status for the FFPE samples was assessed within the framework of a larger study carried out in the Laboratory of Molecular Oncology of the Hellenic Foundation for Cancer Research/Aristotle University of Thessaloniki. A custom panel was developed targeting mutation-relevant coding regions of genes implicated in colorectal carcinoma, including a total of 17 amplicons targeting *KRAS* and *BRAF*, as shown in Additional file 1: Table S1. Amplicon design was based on the GRCh37 (hg19) assembly of the human genome, adapted for FFPE samples (amplicon length up to 175 bp), and resulting primers were assessed for specificity using NCBI’s BLAST tool, while amplicons were evaluated for their position within target genomic regions to include published mutations. FFPE libraries were analyzed in an Ion Torrent Proton Sequencer (Life Technologies/Ion Torrent). Data retrieval, base calling, and the generation of sequence reads were performed on the Torrent Server using Torrent Suite v.5.0.2, followed by adapter sequence trimming, read alignment to the human reference genome, and variant calling. Once variant annotation was performed by Ion Reporter v.5, raw annotated data were evaluated for the reads of all amplicons in the panel (provided by the embedded coverage analysis plug-in) and further quality was filtered with the following eligibility criteria: >100 amplicon reads, variant *P* value <0.0001, variant position coverage >100, variant allele coverage >40, and non-annotated variants and indels involving G-stretches (possibly artifacts with semiconductor sequencing) were excluded. Variant allele frequencies of >5% were accepted by default [18].

Mismatch repair (MMR) status was evaluated on both the primary and metastatic tissue for all CIMP-positive cases. Immunohistochemical nuclear staining was performed for each MLH1 (1:60, Clone ES05; DAKO, Agilent Technologies, Santa Clara, CA), MSH2 (1:30, Clone 25D12, code NCL-MSH2; Novocastra, Leica Biosystems, Wetzlar, Germany), MSH6 (1:70; Clone EP49, code M3646; DAKO), and PMS2 (1:60, Clone M0R4G, code NCL-L-PMS2; Novocastra). The Bond Polymer Refine Detection Kit (Leica Biosystems) was used for staining with 20’citric acid for MLH1 and MSH2 and with 20’EDTA for MSH6 and PMS2. Tumors were considered deficient in expression when there was complete absence of nuclear staining [19]. Cases with at least one protein not expressed were classified as MMR deficient, while cases with intact staining for all four proteins were classified as MMR proficient.

### MethyLight analysis of five CIMP-specific markers in tissue samples

CIMP status in the primary tumor and matched metastases to other organs was determined as described by Weisenberger et al. on a panel of five CRC-specific CIMP markers: *CACNA1G*, *IGF2*, *NEUROG1*, *RUNX3*, and *SOCS1*. We used a methylation-independent *ALUC4* control reaction to normalize input DNA amounts. The percentage of methylated reference (PMR) was calculated as previously described [9]. A marker was considered positive if the PMR was >10. “CIMP-positive” was defined as samples with three or more positive markers and “CIMP-negative” as samples with two or less positive markers. CIMP analysis for each sample was performed twice by two different researchers to ensure the robustness of the results.

The MethyLight PCR reaction mixture consisted of the 2X iTAQ Universal Probes Supermix (BioRad, Hercules, CA) and locus specific primers and probes. The primer and probes were used at final concentrations of 900 and 250 nmol/L, respectively. Bisulfite-converted DNA was used as a template for the MethyLight PCR assay in a final reaction volume of 20 μL. Each MethyLight PCR reaction was performed using the CFX96 Touch™ Real-Time PCR Detection System (BioRad). The thermocycler conditions were 95 °C for 15 min followed by 49 cycles of 95 °C for 15 s and 60 °C for 1 min. One hundred percent of methylated EpiTect Methyl DNA and 100% unmethylated EpiTect Unmethyl DNA (Qiagen, Hilden, Germany) were used as the positive and negative control samples. All samples were run in duplicate for each assay. Data were analyzed using the Bio-Rad CFX manager software version 3.1, and Cq was determined with the Single Threshold method (BioRad).

### Statistical methods

Key clinical variables were compared between primary-metastasis concordance groups, including age, sex, stage, primary site, grade of the primary tumor and the presence of mucinous features, lymphovascular invasion, and/or perineural invasion in the primary tumor. A left-sided colorectal primary was defined as a tumor originating distal to the transverse colon; all other sites were considered right sided.

Continuous variables were presented as median with the corresponding range and categorical variables as frequency with the respective percentages. An ANOVA was used to compare difference in continuous variables between groups, and chi-square or Fisher’s exact tests were used for analyzing categorical variables. SAS software was used for all statistical analyses (SAS Institute Inc., Cary, NC). *P* < 0.05 was considered significant.

## Results

**Fig. 1:**
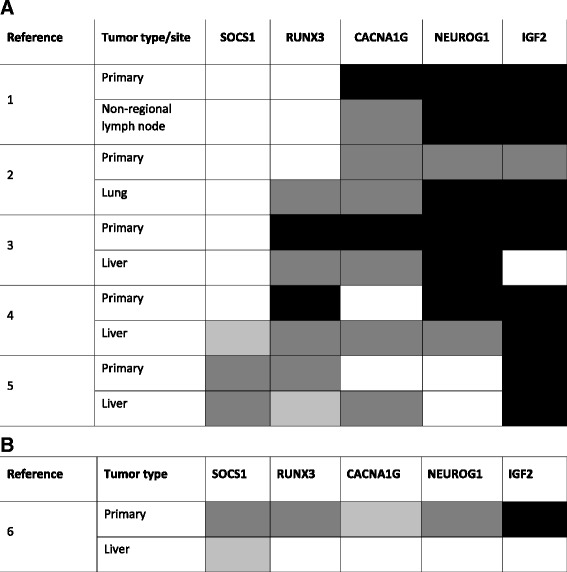
DNA methylation status of a colon cancer CIMP-specific five-gene marker panel in five CIMP-positive primary-metastasis pairs (**a**) and one pair with discordant CIMP status (**b**). Percent methylation reference (PMR) <4% is shown in *white*, 4 to 10% in *light gray*, >10 to 100% in *dark gray*, and >100% in *black*

Of 80 available primary CRC tumors with matched metastases, 70 pairs had sufficient DNA for CIMP determination and were included in the final analysis. Five pairs (7.0%) were CIMP positive both in the primary tumor tissue and in the matched metastasis. One pair was CIMP positive in the primary tumor, but CIMP-negative in the metastasis. The remaining 64 pairs (91.4%) were CIMP-negative in both the primary and metastatic tumors. The concordance is summarized in Table 1. The five pairs with CIMP positivity are shown descriptively in Fig. 1. When we applied a less stringent PMR cut-off value of ≥4%, we observed a slight increase in the number of discordant pairs, with three pairs demonstrating CIMP-negative primary tumors but CIMP-positive matched metastases (Table 2).

**Table 1:** Concordance of CIMP-positivity in primary tumors and matched metastases using a threshold of PMR >10

CIMP PMR >10	Metastasis +	Metastasis −
Primary +	5 (7.1%)	1 (1.4%)
Primary −	0	64 (91.4%)

**Table 2:** Concordance of CIMP-positivity in primary tumors and matched metastases using a threshold of PMR >4

CIMP PMR >4	Metastasis +	Metastasis −
Primary +	6 (8.6%)	1 (1.4%)
Primary −	3 (4.3%)	60 (85.7%)

**Fig. 2:**
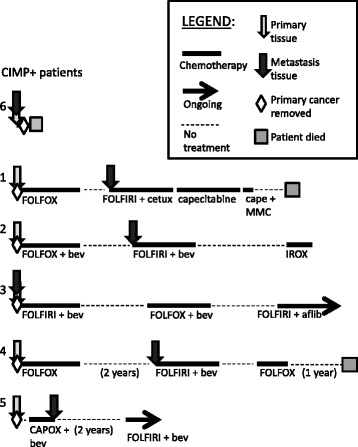
Visual description of the treatment history for each of the CIMP-positive patients. Abbreviations: *FOLFOX* 5-fluorouracil (5-FU), leucovorin (LV), and oxaliplatin, *IROX* irinotecan, oxaliplatin, *FOLFIRI* 5-FU, LV, and irinotecan, *cetux* cetuximab, *cape* capecitabine, *MMC* mitomycin C, *bev* bevacizumab, *CAPOX* capecitabine, LV, oxaliplatin, *alfib* aflibercept

The patient demographics and sample characteristics are presented in Table 3. There were no statistically significant differences in the age, sex, stage at diagnosis, primary site, or analyzed histopathological features between any of the groups (concordant CIMP-positive cases vs. concordant CIMP-negative cases). The majority of case pairs were stage IV at diagnosis (i.e., synchronous metastases; 69%) and right sided (79%). For metachronous cases, the mean time to detection of metastatic disease was 1.72 years (range 0.40–6.61). Among patients with CIMP-positive tumors, there was no obvious association between timing of metastatic disease (i.e., synchronous vs. metachronous) and treatment. Four of the five concordant CIMP-positive patients received an oxaliplatin-based chemotherapy regimen prior to sampling of the metastatic tumor tissue. The individual details of the six patients with CIMP-positive tumors are shown in Table 4. Of the CIMP-positive CRC cases, four patients (67%) were male and five (83%) had left-sided tumors. Notably, no *BRAF* mutations were detected in any of the primary tumors or matched metastases, despite the fact that the corresponding amplicons were read at adequate depth. In one CIMP-positive patient, a *KRAS* p.G12D mutation was detected in both the primary tumor and matched liver metastasis; in the patient with discordant CIMP status, the primary tumor carried *KRAS* p.Q61H. None of the CIMP-positive cases were found to have MMR deficiency. The treatment history for these six patients CIMP-positive tumors is visually described in Fig. 2.

**Table 3:** Clinical variables of interest and CIMP concordance status

Variable	Metastasis − primary− (*n* = 64)		Metastasis − primary+ (*n* = 1)		Metastasis + primary− (*n* = 0)		Metastasis + primary+ (*n* = 5)		*P value
	*N*	%	*N*	%	*N*	%	*N*	%	
Age									0.52
Mean (range)	61.0	[24.2, 79.9]	74.4	[74.4, 74.4]			60.6	[51.1, 66.8]	
Missing	8		0		0		0		
Sex									0.36
Female	30	50%	1	100%			1	20%	
Male	30	50%	0	0			4	80%	
Missing	4		0		0		0		
Stage at diagnosis									>0.99
I	0	0	0	0			0	0	
II	6	11%	0	0			0	0	
III	13	23%	0	0			1	20%	
IV	37	67%	1	100%			4	80%	
Missing	7		0		0		0		
Primary site									>0.99
Right	12	21%	0	0			1	20%	
Left	45	79%	1	100%			4	80%	
Missing	7		0		0		0		
Histological grade									0.21
Grade 1–2	35	66%	1	100%			5	100%	
Grade 3	18	34%	0	0			0	0	
Missing	11		0		0		0		
Mucinous features									0.10
No	45	82%	1	100%			2	40%	
Yes	10	18%	0	0			3	60%	
Missing	9		0		0		0		
Lymphovascular invasion									>0.99
No	38	72%	1	100%			4	80%	
Yes	15	28%	0	0			1	20%	
Missing	11		0		0		0		
Perineural invasion									>0.99
No	40	71%	1	100%			4	80%	
Yes	12	21%	0	0			1	20%	
Not applicable	4	7%	0	0			0	0	
Missing	8		0		0		0		
Molecular status, mutant									
*KRAS*	22	37%	1	100%	0		1	20%	0.33
*BRAF*	2	3%	0	0	0		0	0	>0.99
*NRAS*	2	3%	0	0	0		0	0	>0.99
Mismatch repair status, deficient^a^			0	0	0		0	0	n/a

*P values for categorical variables calculated with a Fisher’s exact test. *P* values for continuous variables calculated with an ANOVA

^a^Mismatch repair status was performed for cases with detected CIMP positivity in the primary and/or metastatic tissue

**Table 4:** Individual clinical characteristics of patients with CIMP-positive cancers

Patient number	Age	Sex	Stage at diagnosis	Primary site	Metastatic site	Histologic grade	Mucinous features	Lymphovascular invasion	Perineural invasion	KRAS	BRAF	MMR status
1	62	Male	IV	Left	Non-regional node	2	Yes	Yes	No	Wild-type	Wild-type	Proficient
2	62	Male	IV	Left	Lung	2	Yes	No	No	Wild-type	Wild-type	Proficient
3	60	Male	IV	Left	Liver	2	No	No	No	Wild-type	Wild-type	Proficient
4	66	Male	III	Right	Liver	2	Yes	No	No	Mutant	Wild-type	Proficient
5	51	Female	IV	Left	Liver	2	No	No	Yes	Wild-type	Wild-type	Proficient
6	75	Female	IV	Left	Liver	2	Yes	Yes	No	Mutant	Wild-type	Proficient

## Discussion

In this paper, we determined the CIMP status in 70 pairs of primary CRC and matched metastases using a validated CRC marker panel [9]. Using PMR >10% as a cut-off value to score a gene as methylated, we found that CIMP status is concordant in the great majority (98.6%) of the pairs. This finding indicates that in CRC, CIMP status in primary tumors reflects the CIMP status of the metastasis, regardless of the site of metastasis. The clinical implication of this result is that by determining the CIMP status in primary tumors, one can have confidence in predicting the CIMP status in disseminated metastatic lesions even when they are not directly assayed. Using the same five-gene CIMP panel and definitions, Messick et al. reported loss of CIMP in lymph node metastases in more than half of the 13 CIMP-positive primary CRCs [20]. However, we observed loss of CIMP positivity in only one case of liver metastasis out of the 6 CIMP-positive primary-metastasis pairs. The reported differences in the concordance between primary and matched metastases could reflect tissue heterogeneity, differences in the site of metastatic lesions, or differences in the patient populations in the two studies. It is noteworthy that when we applied a less stringent PMR cut-off value of 4%, we observed a slight increase in the number of discordant pairs, with 3 pairs demonstrating CIMP-negative primary tumors but CIMP-positive matched metastases (Table 2).

Our limited sample size of CIMP CRCs (*n* = 1 of discordant pairs using PMR >10%) does not allow us to distinguish the possible reasons for the discordance of CIMP between primary and metastatic tumor lesions. A larger study including more CIMP-positive cases is needed to confirm our results and evaluate possible mechanisms for CIMP discordance in those situations where that does occur. In our case, the primary was positive in 4/5 markers, while the metastasis had no evidence of methylation in any of the markers, suggesting loss of the aberrant methylation. Due to small numbers, no inferences could be made about the impact of chemotherapy treatment on CIMP positivity.

Although CIMP has been recognized as a distinct molecular subgroup in CRC, the biological significance of CIMP during colon cancer development and progression is not well understood. It is known that aberrant methylation occurs early in the pre-malignant (polyp) stage and, in fact, most CIMP-positive polyps progress through an alternative pathway originating from sessile serrated polyps that can ultimately result in CIMP-positive CRCs [21, 22]. However, it is not clear whether epigenetic alterations, such as CIMP, can be acquired or lost in metastatic cancer cells that disseminate from primary cancers or whether CIMP is heterogeneous in primary tumors, which could result in metastases that have discordant CIMP status from the site sampled in the primary tumor. In this study, we observed that 83.3% of matched metastatic lesions were CIMP-positive if the primary tumor was CIMP-positive. Although our study has a small number of CIMP-positive CRCs, our findings suggest that CIMP is maintained through primary tumor development and metastatic progression, possibly secondary to a growth advantage in CIMP-positive cancer cells resulting from CpG island methylation-mediated transcriptional silencing of key tumor suppressor genes. Of note, the liver metastasis that lost CIMP positivity from the primary tumor was a synchronous site of disease, indicating the epigenetic heterogeneity between the primary tumor and metastatic lesions existed at the time of diagnosis. Supporting the concept of clonal heterogeneity, the discordant CIMP case also had discordant *KRAS* mutation status. In addition, chemotherapy exposure did not appear to affect CIMP positivity in metachronous metastases.

In our study, we identified 7.8% CIMP-positive samples in 140 samples, lower than the observed frequency of 15–20% reported in the USA [13]. The difference in the observed frequency of CIMP-positive cases in various studies is possibly due to the different CIMP gene panels and assays used in each study and differences in study populations [23]. While limited by small numbers, the CIMP-positive group did not fit the standard demographics [9]. There was a predominance of male patients with left-sided *BRAF*-wild-type tumors, while two of our six CIMP-positive tumors had mutations in *KRAS*, which are reported at low incidence in a methylator environment [24]. The Greek patient population, the Mediterranean diet, or differences in environmental exposures might account for the low CIMP frequency, lower frequency of *BRAF* mutations, and different patient demographics in our sample set [25]. In addition, as all patients in the study were metastatic, this may have contributed to a slightly different molecular profile than CIMP cancers overall. For example, most of the CIMP-positive pairs had liver metastases, the primary tumors of which have been reported to be CIMP and *BRAF*-mutation poor [26]. The fact that the majority of the cases were stage IV CRC is also likely responsible for the observed lower frequency of MMR deficiency, a surrogate for MSI.

## Conclusions

In conclusion, despite the relatively small number of CIMP-positive cases identified in the study, we found strong concordance of CIMP status between primary CRC and matched metastasis. Thus, we suggest that CIMP status in the primary tumor should serve as a reliable biomarker across all stages of disease and at different stages of treatment and can, therefore, be used to predict the CIMP status of metastatic disease.

## Additional file

Additional file 1: Table S1. Amplicons targeting *KRAS* and *BRAF*, within the IAD47763_031 custom panel. (DOCX 17 kb)

## Abbreviations

5-FU: 5-Fluorouracil
CIMP: CpG island methylator phenotype
CIN: Chromosomal instability
CRC: Colorectal cancer
MMR: Mismatch repair
MSI: Microsatellite instability
MSS: Microsatellite stable
PMR: Percent methylated reference
