# Supplementary material for: The CpG island methylator phenotype is concordant between primary colorectal carcinoma and matched distant metastases
Source: Clin Epigenetics. 2017 May 2;9:46. doi: 10.1186/s13148-017-0347-1 (PMC5414304; doi:10.1186/s13148-017-0347-1)
Supplement: Additional file 1: Table S1. — Amplicons targeting KRAS and BRAF, within the IAD47763_031 custom panel. (DOCX 17 kb) [file 13148_2017_347_MOESM1_ESM.docx]

**Additional file 1: Table S1. Amplicons targeting KRAS and BRAF, within the IAD47763_031 custom panel.**

| **Gene** | **Chromo-some band** | **Location within gene** | **Amplicon start*** | **Amplicon stop*** | **Ion AmpliSeq Forward primer*** | **Ion AmpliSeq Reverse primer*** | **Insert size** |
| --- | --- | --- | --- | --- | --- | --- | --- |
| BRAF | 7q34 | in15-ex15 | 140452989 | 140453114 | GTTGAGACCTTCAATGACTTTCTAGTAACT | CCATCAGTTTGAACAGTTGTCTGGAT | 69 |
| BRAF | 7q34 | ex15 | 140453061 | 140453221 | GCCTCAATTCTTACCATCCACAAAATG | CTGTTTTCCTTTACTTACTACACCTCAGA | 104 |
| BRAF | 7q34 | ex15-in14 | 140453135 | 140453280 | CACTGTAGCTAGACCAAAATCACCTATT | AATTAGATCTCTTACCTAAACTCTTCATAATGC | 84 |
| BRAF | 7q34 | in11 | 140481224 | 140481388 | GTTAGAAACTTTTGGAGGAGTCCTGA | GGAAAGTGGCATGGTAAGTATGTAATGT | 110 |
| BRAF | 7q34 | in11-ex11 | 140481333 | 140481458 | TCCTATTATGACTTGTCACAATGTCACC | GGAGATTCCTGATGGGCAGATTA | 74 |
| BRAF | 7q34 | ex11-in10 | 140481412 | 140481579 | CAGATCCAATTCTTTGTCCCACTG | AGGCATAAGGTAATGTACTTAGGGTGAA | 115 |
| KRAS | 12p12.1 | in5-ex5 | 25368298 | 25368463 | AGTGGTTGCCACCTTGTTACC | GGGAGATCCGACAATACAGATTGAAAAA | 116 |
| KRAS | 12p12.1 | ex5-in4 | 25368409 | 25368560 | CCAGGAGTCTTTTCTTCTTTGCTGATT | GGCTTTCCCAGTAAATTACTCTTACCA | 97 |
| KRAS | 12p12.1 | in4 | 25378397 | 25378560 | GAAGCAATGCCCTCTCAAGAGA | AAAGACAAGACAGGTAAGTAACACTGAAA | 112 |
| KRAS | 12p12.1 | in4-ex4 | 25378487 | 25378613 | AATGACATAACAGTTATGATTTTGCAGAAAA | CAGGCTCAGGACTTAGCAAGAAG | 72 |
| KRAS | 12p12.1 | in3-ex3 | 25380135 | 25380262 | AATGTCAGCTTATTATATTCAATTTAAACCCAC | GCAATGAGGGACCAGTACATGA | 72 |
| KRAS | 12p12.1 | ex3 | 25380223 | 25380348 | GAAAGCCCTCCCCAGTCC | AGGATTCCTACAGGAAGCAAGTAGT | 82 |
| KRAS | 12p12.1 | ex3-in2 | 25380269 | 25380397 | CTCCTCTTGACCTGCTGTGT | TGAAGTAAAAGGTGCACTGTAATAATCCA | 79 |
| KRAS | 12p12.1 | in2 | 25398026 | 25398177 | AGGAAAGTAAAGTTCCCATATTAATGGTT | GTGCAGGACCATTCTTTGATACAGATA | 95 |
| KRAS | 12p12.1 | in2-ex2 | 25398122 | 25398285 | GTACTCATGAAAATGGTCAGAGAAACCT | GGTGGCGTAGGCAAGAGTG | 116 |
| KRAS | 12p12.1 | ex2-in1 | 25398239 | 25398393 | TGATTCTGAATTAGCTGTATCGTCAAGG | AGTGTATTAACCTTATGTGTGACATGTT | 98 |
| KRAS | 12p12.1 | ex1-5'UTR | 25403752 | 25403900 | TCGCTCCCAGTCCGAAATG | GCCCCGAACTCATCGGT | 112 |

***: GRCh37**
